# Barriers to glucagon-like peptide-1 agonists used for obesity management among the general population in Tabuk City, Saudi Arabia, and their relation to smoking cessation and antidepressants

**DOI:** 10.3389/fphar.2025.1510554

**Published:** 2025-07-31

**Authors:** Amirah Alhowiti, Hyder Mirghani, Abdulaziz Abdulrahman Qrmli, Amal Abdullah Albalawi, Raneem Abdulrahman Aljohani

**Affiliations:** ^1^ Department of Family and Community Medicine, Faculty of Medicine, University of Tabuk, Tabuk, Saudi Arabia; ^2^ Department of Internal Medicine, Faculty of Medicine, University of Tabuk, Tabuk, Saudi Arabia

**Keywords:** obesity, barriers, GLP-1 agonists, nicotine, antidepressant use

## Abstract

**Background:**

Obesity is a chronic inflammatory disease with high morbidity that is decidedly prevalent worldwide and in Saudi Arabia. Glucagon-like peptide 1 receptor agonists (GLP-1 agonists) are broadly used for the management of diabetes and obesity. We aimed to assess barriers to GLP-1 agonist use among the general population in Saudi Arabia and their association with smoking and antidepressants use.

**Methods:**

This cross-sectional study was conducted in Tabuk, Saudi Arabia from January to October 2024 using a structured questionnaire based on age, sex, lifestyle, GLP-1 agonists, antidepressant use, source of information regarding GLP-1 agonists, type of injection, smoking status, and whether smoking frequency/amount decreased following GLP-1 agonists use.

**Results:**

out of the 481 participants, 28.4%, and 30.7% were on regular exercise and a healthy diet respectively, and 21.8% were using GLP-1 agonists of them, 54.5%, and 42.7% interrupted their GLP-1 agonists due to shortage/cost and side effects. Semaglutide was the most common GLP-1 agonist used (13.7%). No significant associations were found between GLP-1 agonist use, age, smoking, and depression (odds ratio, 0.978, 1.073, and 0.770, respectively). A significant association was found with gender, BMI, diet, and exercise.

**Conclusion:**

GLP-1 agonist uptake was relatively low, the majority of patients used GLP-1 agonists for weight reduction and not for comorbidities. More than half interrupted their intake due to cost/unavailability. Semaglutide was the most commonly prescribed medication. No significant associations were found between GLP-1 agonists use, age, smoking, and antidepressant medication use, a significant association was found with females, BMI, diet, and exercise.

## Introduction

Diabetes and obesity are major health concerns with diabetes mellitus affecting 10.5% globally and expected to increase by 21.1% in middle-income countries and 12.1% in high-income countries by 2045 (Sun et al., 2022). Importantly, a growing trend of obesity and diabetes was observed in nearly all countries due to global urbanization, unhealthy diets, high body mass index, and increasing income. One billion are expected to live with obesity by the year 2030 according to the World Obesity Federation (Lovic et al., 2020). The situation in Saudi Arabia is alarming because 35% of the general population is obese, and 75.2% are either obese or overweight. In addition, Saudi Arabia is in the diabetes super-region with a prevalence of 18.1% according to the Diabetes International Federation and the real data could be higher (Sun et al., 2022; World Obesity Federation, 2024). Therefore, urgent, effective, and preventive treatment strategies are needed for obesity (Alfaris, 2021).

Obesity is a chronic inflammatory disease like diabetes, dyslipidemia, and high blood pressure. However, it is usually ignored and undertreated with increasing comorbidity and mortality (Washington et al., 2023). Despite the above, healthcare personnel felt that patients with obesity lack interest and are not motivated to lose weight (Tran et al., 2025). The significant barriers to the approved anti-obesity drugs are concerns regarding side effects, cost, and effectiveness. Another important barrier is the lack of insurance coverage (Kaplan et al., 2025). A study conducted in Malaysia found that only 10% of patients with obesity received pharmacological treatment due to the physician’s time constraints, limited awareness of treatment options, and lack of motivation (Nor et al., 2025). In the Kingdom of Saudi Arabia, the knowledge is limited regarding weight management medications (Algarni et al., 2023). Importantly, 73% of physicians never prescribed antiobesity medications and 90% of general practitioners are not familiar with antiobesity medication indications (Alshammari Al-Shammari Yf, 2014; Al-Khaldi et al., 2014).

GLP-1 agonists use among patients with type 2 diabetes started with the approval of the twice/daily exenatide in 2005. Many classes were developed in the following years. Semaglutide once/week subcutaneous injection was approved in 2017 for glycemic control, and 2021 for the treatment of obesity (Bzow and yckyj, 2020; Wilding et al., 2021).

Obesity and diabetes relationship is bidirectional and when co-existing exacerbates each other’s lethal comorbidities including high blood pressure, metabolic-associated fatty liver disease, and cancer (Kloock et al., 2023). There is an increasing awareness of body weight management in cardiovascular risk factors prevention, and comorbidity-oriented metabolic surgery.

GLP-1 agonists development has revolutionized the treatment of obesity and diabetes mellitus, animal studies showed that tirzepatide (a dual GLP-1/GIP-agonist) is superior to bariatric surgery in weight reduction and diabetes remission (Courcoulas et al., 2024; Al-Sabah et al., 2024). While randomized trials with long follow-up periods showed that bariatric surgery for diabetes remission is superior to lifestyle/medications (Diallo et al., 2023). However, high doses of GLP-1 agonists are associated with gastrointestinal side effects, while bariatric surgery is invasive and some bariatric surgery procedures are irreversible (K et al., 2024). Bariatric surgery is recommended for obese patients and those with a body mass index of >27 and a presence of comorbidities. However, only 1.9 percent of patients undergo surgery with substantial region variations (Dixon, 2016).

The effects of GLP-1 agonists on depression are a matter of controversy (Chen et al., 2024). Importantly, recent reports emphasized liraglutide and semaglutide are associated with depression, suicidal thoughts, and self-injury (EMA, 2025). In the case of semaglutide, depression, suicidal thoughts, and self-injury; a warning had already been made explicit for Wegovy but not for Ozempic (FDA, 2025a; FDA, 2025b). Although several issues are still unclear (FDA, 2025c), EMA recently decided to closely monitor the issue, especially with certain GLP-1 agonists formulations (FDA, 2025b).

The observation of suddenly quitting smoking in patients prescribed GLP-1-like receptor agonists, and the previous animal studies suggest that the association between GLP-1 agonists and nicotine-seeking behavior reduction should not go unnoticed (Rubin, 2024). Therefore, GLP-1 agonists use can be extended to include nicotine use dependence in particular among patients with diabetes and high body mass index.

The effects of GLP-1 agonists on smoking cessation were discussed controversially, some studies reported the positive effects of Exenatide and semaglutide on ending smoking, however, Exenatide effects were reported in particular among patients with mild depression, and those who smoke >20 cigarettes/day (Yammine et al., 2025; Popovic et al., 2024). On the other hand, Lengsfeld et al. (2023) conducted a trial and showed no effects of dulaglutide on abstinence rates.

Smoking is a major global health concern, the rising popularity of e-cigarettes or electronic delivery significantly increases traditional smoking because e-cigarette users are 3.5 times more likely to use traditional smoking than their counterparts (Herman and Schmidt, 2024; Soneji et al., 2017). Although quitting smoking at any age significantly reduces morbidity and mortality, more than two-thirds of adult smokers, and 65.3% of adolescents want to quit. However, only one in ten attempts were successful due to withdrawal and craving symptoms (Taylor et al., 2002; Dai, 2021).

Obesity is associated with fatal complications including insulin resistance, diabetes, dyslipidemia, hypertension, obstructive sleep apnea, and certain malignancies. Though data about obesity management in Saudi Arabia is limited and uptake of obesity medications is minimal. Therefore, this study aimed to investigate barriers to GLP-1 agonists used for obesity management among the general population in Tabuk City, Saudi Arabia. In addition, the association between GLP-1 agonists smoking and antidepressant use was assessed.

## Subjects and methods

This cross-sectional study was conducted in Tabuk City, Saudi Arabia from January 2024 to October 2024. A multi-stage sampling technique was used to collect the participants. The information was taken from the Saudi Authority, then a map was formed in which Tabuk City was divided into five areas, then five big Malls were randomly selected to recruit the participants. All adults who agreed to participate were approached (consecutively). The researchers measured the weight and height of the participants, and their contact details were taken to send the questionnaire.

### Inclusion and exclusion criteria

All adult people in Tabuk City above 18 years were eligible. Children and pregnant women were excluded from the study.

### Sample size calculation

We assumed that the prevalence of obesity is unknown in Tabuk because of the lack of National Surveys, therefore, the sample size was calculated at 5% marginal error and 50% prevalence, the total population in Tabuk City is 760000 according to 2021 data. The sample size was 385.

### Data collection procedure

A structured questionnaire based on age, sex, level of exercise (regular, irregular, and no exercise), if on a healthy diet with high fruits and vegetables, high complex carbohydrates, and low refined carbohydrates, if prescribed GLP-1 agonists, source of information regarding GLP-1 agonists, and type of injection (semaglutide, liraglutide, or tirzepaptide). Smoking status was evaluated by asking about smoking, frequency, and amount and whether smoking decreased in frequency and amount following GLP-1 agonists use. The questionnaire inquired about medications use including oral contraceptives and antidepressant use (have you ever been prescribed medications for depression?). Patients on GLP-1 agonists were asked about the reason behind GLP-1 agonists use (weight reduction/chronic disease risk reduction), the use of GLP-1 agonists (regular or interrupted), and the reason behind discontinuation (cost/inaccessibility, side effects, and cosmetic reasons). Smoking, oral contraceptive use, and antidepressant medications were reported. The questionnaire inquired on knowledge about Ozempic (semaglutide) face, which is accelerated facial and skin aging, and a history of cosmetic surgery. The body mass index before and after GLP-1 agonists use was reported by the researchers. The questionnaire was distributed in the Arabic language, and approved by an English/Arabic expert, a Family Physician, and an Endocrinologist and the respondents were provided with the researcher’s contact details for any difficulty in filling out the questionnaire.

### Ethical considerations

The Declaration of Helsinki was strictly followed for the management of the data collected, the patient’s privacy was ensured, and no personal information was recorded. Anonymization procedures were adopted, and the participants were informed about the study’s purpose and their right to withdraw. The first question gave consent to fill out the questionnaire, if the respondent chose not to agree, he/she would automatically be cut from the survey. The University of Tabuk, Saudi Arabia approved the research on February 4, 2024, reference number (UT-336-175-2024).

### Data analysis

The Statistical Package for Social Science (IBM, version 20, New York) was used for the data analysis. The data were presented as percentages, and mean ± standard deviation, and a sub-analysis was conducted among those on GLP-1 agonists (n = 105). The Binary Logistic Regression Analysis was used to assess the relationship between GLP-1 agonists use, age, gender, diet, exercise, BMI, smoking, and antidepressant medications use, The (B, 1.44), Exp (B), 4.237, and P-values were reported. A P-value of <0.05 was considered significant.

## Results

### Characteristics of the participants

The study included 481 patients (55.7% women), their ages, 31.79 ± 9.31 years, 43.9% had previously been diagnosed with obesity, 28.4%, and 30.7% were on regular exercise and a healthy diet respectively, while 21.8% were using glucagon-like peptide-1 receptors agonists (GLP-1 agonists), 11.2% conducted bariatric surgery (some patients used > one obesity management). In this study, 25.3% of the total sample were smoking cigarettes and smoking decreased by 3.5% following GLP-1 agonists use. In the present study, 13.1% were on oral contraceptive pills, and 5.8% were on antidepressant medications. Table 1.

**TABLE 1:** Basic characteristics of the study group.

Age/years (range 36-82)	31.79 ± 9.31
Gender	
Women	268 (55.7%)
Men	213 (44.3%)
Previously diagnosed with obesity	211 (43.9%)
Exercise	
Regular exercise	137 (28.4%)
Irregular exercise	251 (52.1)
No exercise	94 (19.5%)
On healthy diet	148 (30.7%)
Bariatric surgery	24 (11.2%)
GLP-1 agonists use	105 (21.8%)
Have heard about Ozempic face	87 (18.1%)
Smoking	122 (25.3%)
Smoking decreased in frequency/amount after GLP-1 agonists	017 (3.5%)
On oral contraceptives (number = 268)	35 (13.1%)
On antidepressant Medications	28 (5.8%)

### Sub-analysis in patients on GLP-1 agonists only (n = 105)

Table 2 shows the findings in patients who used GLP-1 agonists (total number 105). The findings were as follows: 72.4% of patients used GLP-1 agonists for weight reduction, 27.6% used them for comorbidities reduction, 54.5% of the participants interrupted their GLP-1 agonists, of those who interrupted GLP-1 agonists, shortage/cost was the most common reason for GLP-1 agonists interruption and reported in 43.8%, side effects in 42.7%, while 13.3% stopped GLP-1 agonists due to cosmetic reasons. Importantly, the body mass index reduced from 34.1 ± 9 to 30.6 ± 6, following GLP-1 agonists use. Interestingly, only one in five (21.2%) advised for GLP-1 use, 29.8% advised against it, while 49% were neutral. In this study, 24.03% of patients on GLP-1 agonists used to smoke cigarettes, and smoking decreased by 14.4% following GLP-1 agonists use. Interestingly, 10.5% were on antidepressant medications, and 7.7% were on oral contraceptives, while 6.7% underwent facial cosmetic surgery. Table 2.

**TABLE 2:** The pattern of GLP-1 agonists use among adults in Tabuk City, Saudi Arabia (total 105).

Character	No%
What is the purpose behind GLP-1 agonists use? (no = 105)	No%
Weight reduction	76 (72.4%)
Chronic disease risk reduction	29 (27.6%)
Use of GLP-1 agonists (no = 105)	
Regular	48 (45.5%)
Interrupted	57 (54.5%)
The reason behind drug discontinuation of GLP-1 agonists (no = 105)	
Side effects	46 (43.8%)
Shortage/cost	45 (42.7%)
Cosmetic reasons	14 (13.3%)
Advice other to taking GLP-1 agonists use (no = 105)	
Yes	22 (21.2%)
No	31 (29.8%)
Neutral	52 (49%)
Smoking status (no = 105)	
Smoking	25 (24.03%)
Smoking decreased in frequency/amount after GLP-1 agonists use	015 (14.4%)
On oral contraceptives (no = 105)	08 (7.7%)
On antidepressant medications (no = 105)	11 (10.5%)
Face cosmetic surgery (no = 105)	07 (6.7%)
Body mass index before GLP-1agonists	34.1 ± 9
Body mass index after GLP-1 agonists	30.6 ± 6

Regarding the association between GLP-1 agonists association with various characters, we used the Binary Logistic Regression Analysis. A significant negative association was found regarding body mass index (B, −0.180), Exp (B), 0.835, and P-value <0. 001, gender, (B, 1.44), Exp (B), 4.237, and P-value <0. 001, diet (B, −864), EX = Ex (B), 0.421, and P-value, 0.001, and exercise, and (B, −0.812), Exp (B), 0.441, and P-value <0. 001. No association was evident regarding GLP-1 agonists use, age of the participants (B, −0.23), Exp (B), 0.978, and P-value, 0.205 smoking, (B, −0.262), Exp (B), 0.770, and P-value, 0. 596, and antidepressant medications use (B, 0.071), Exp (B), 1.073, and P-value, 0. 447. Table 3.

**TABLE 3:** The relationship between GLP-1 agonists uses, age, gender, body mass index, smoking, and antidepressant use among obese patients on/not on GLP-1 agonists in Saudi Arabia.

Character	B	Odd ratio	P-value
Age	−0.023	0.978	0.205
Smoking	−0.262	0.770	0.569
Antidepressants use	0.071	1.073	0.447
Body mass index	−0.180	0.835	<0.001
Gender	1.44	4.237	<0.001
Diet	−0.864	0.421	0.001
Exercise	−0.812	0.441	0.004
Constant	5.570	262.455	<0.001

*Binary Logisti Regression Analysis.

**FIGURE 1:**
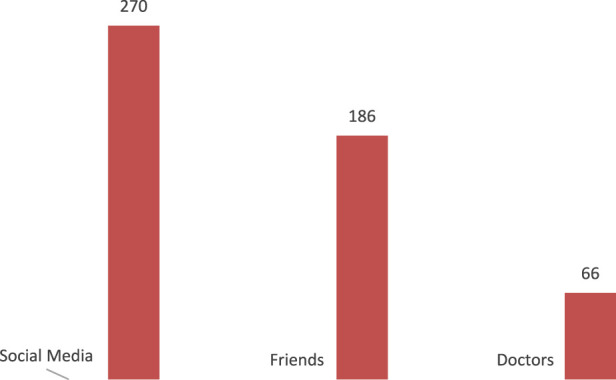
Source of information regarding GLP-1 agonists.

The majority of participants have heard about GLP-1 agonists through social media and friends (56.1% and 38.7% respectively), and only a minority received the knowledge from doctors (13.7%.) Figure 1.

**FIGURE 2:**
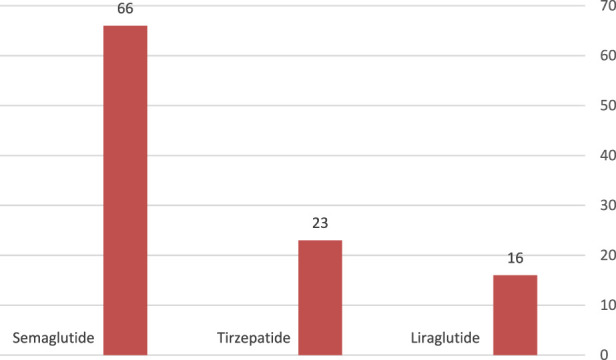
Type of GLP-1 agonists.

Semaglutide was the commonest GLP-1 agonist used (13.7%), terzipatide was used in 4.8%, while 3.3% of patients with high body mass index were on liraglutide. Figure 2.

**FIGURE 3:**
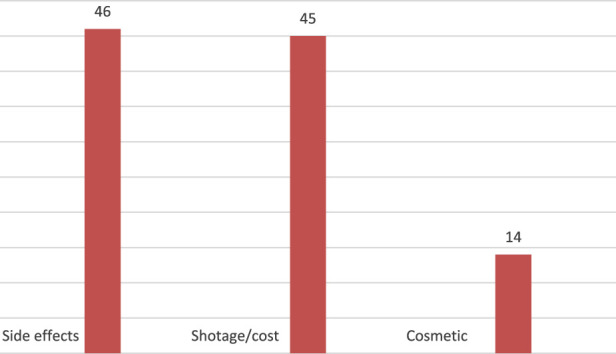
Reasons behind GLP-1 agonists discontinuation.

Shortage/cost was the most common reason for GLP-1 agonists interruption and reported in 43.8%, side effects in 42.7%, while 13.3% stopped GLP-1 agonists due to cosmetic reasons. Figure 3.

## Discussion

The majority of smokers who quit smoking gain weight (some gain more than 20 pounds) due to the increased consumption of high-palatable food, the transient weight gain can increase the risk of cardiovascular risk factors including diabetes and hypertension and prevent improvement in lung function (Soule et al., 2020; Gentzke et al., 2022; Audrain-McGovern and Benowitz, 2011). The above observations apply to both traditional and e-cigarettes among all age groups.

Many drugs are approved for nicotine use dependence with modest efficacy including Bupropion, varenicline, and nicotine replacement therapy, however, they do not prevent weight gain following abstinence (Bush et al., 2016). Because of the above, drugs targeting both cravings for food during abstinence and body weight are attractive. The FDA approved GLP-1 agonists for obesity and type 2 diabetes treatment with mounting evidence in reducing the reward effects and hyperphagia-induced body weight gain following illicit drugs and nicotine quitting (Chinn et al., 2005; Mills et al., 2012; Davies et al., 2021; Herman et al., 2023).

Recent animal studies showed that GLP-1 agonists reduce nicotine-induced taking- and seeking behaviors, in addition, these novel medications attenuate withdrawal-induced hyperphagia and body weight gain following nicotine abstinence (Tuesta et al., 2017). Studies on humans showed conflicting results, some studies reported the positive effects of Exenatide and semaglutide on smoking cessation (Yammine et al., 2025; Popovic et al., 2024). While a study on dulaglutide showed no effects on abstinence rates (Lengsfeld et al., 2023).

In the present study, smoking was reported in 24.03% of participants, which is slightly higher than studies conducted in the adult Saudi population, in which 19.4% and 17.3% in the southern and central regions were current smokers (AlBariqi et al., 2025; Nasser et al., 2025).

In the present study, no differences were found between patients taking GLP-1 agonists and their counterparts regarding smoking rates, odds ratio of 0.770. Our findings were not in line with Yammine et al. (2021) who conducted a pilot study with a limited number of patients and found that Exenatide 2 mg reduced craving and post-nicotine cessation weight gain. However, the authors combined Exenatide with nicotine replacement therapy. The discrepancy between the results could be explained by the differences in the sample size, medications used for obesity, the questionnaires, and the different populations.

In the present study, 5.8% of participants were on antidepressant medications compared to 12.7% in the Saudi general population, the lower rate could be explained by the fact that some physicians were concerned about GLP-1 agonists prescription to patients with depression due to the reported association with suicidality (Alhabeeb et al., 2023; Sam et al., 2011).

The effects of GLP-1 agonists on depression are a matter of controversy (Chen et al., 2024). Our data showed no difference between patients on GLP-1 agonists and those on lifestyle regarding the use of antidepressant medication, odds ratio of 1.073.

Importantly, recent reports emphasized liraglutide and semaglutide GLP-1 agonists-associated risk of triggering depression, suicidal thoughts, and self-injury (EMA, 2025). In the case of semaglutide, depression, suicidal thoughts, and self-injury, a warning had already been made explicit for Wegovy but not for Ozempic (FDA, 2025a; FDA, 2025b). Although several issues are still unclear (FDA, 2025c), EMA recently decided to closely monitor the issue, especially with certain GLP-1 agonists formulations (FDA, 2025b).

In the present study, 30.7% were on a healthy diet, regular activity was reported in 28.4%, and 21.8% of patients were prescribed GLP-1 agonists, while bariatric surgery was conducted in 4.9% of patients. The current observation was similar to previous studies in Canada which reported that 9.2% of patients with obesity received obesity therapy under medical supervision (Patton et al., 2023). A study conducted in the United States (Garvey et al., 2024) found that 49% of patients with obesity were prescribed antiobesity medications, similar to the current observations. The low rate of healthy lifestyles among this sample aligns with a previous study by Tham and colleagues who conducted a survey in nine Asian countries and pointed toward unhealthy lifestyles as the major barriers to obesity management (Tham et al., 2024). In the current study, no association between GLP-1 agonists use, healthy diet, and physical activity. In addtion, the rate of healthy lifestyle is inadequate. Therefore, counseling about healthy diet, and physical activity is crucial for the long term weight maintainance (Teicholz et al., 2025). Increasing the quantity (1.4-2.4 g/kg body weight) and quality of proteins (high leucine diet) toghether with exercise help to maintain lean body mass and muscle mass during rapid weight loss (Sforzo et al., 2024). Importantly, weight cycling is associated with cardiometabolic diseases including diabetes, heart failure, and obstructive sleep apnea, because of that healthy lifestyle, and adherence to GLP-1 agonists are curtial to avoid the lethal consequences (Chianelli et al., 2023).

The cost was a major barrier to GLP-1 agonist uptake in the United States (72%-82% are concerned about GLP-1 agonists) the percentage is higher than the current findings in which 42.7% of patients felt that cost/shortage are barriers to GLP-1 agonists reimbursement (Holland and Tiggemann, 2016). A plausible examination might be that GLP-1 agonists are available for free to patients with diabetes and obesity in Saudi Arabia. Importantly, the use of GLP-1 agonists was not regular in 54.5% of patients with obesity, and the primary goal of the treatment is to reduce weight rather than to prevent obesity-related comorbidities (72.4% versus 27.6%). Plausible explanations could be the lack of awareness that obesity is a chronic disease requiring long term antiobesity medications and patients are concerned about weight loss (the idea of thinness within social media), not comorbidities (Garvey et al., 2016). Cultural perceptions and physician prescribing patterns are other possible explanations (Washington et al., 2023). Therefore, increasing awareness is highly needed because the American Association of Clinical Endocrinologists clinical guidelines emphasize that a complications and morbidities-centered approach should be the most desired (Aitken, 2016). Saudi Arabia is a high-income country with high expenditure on diabetes and its complications (25 billion spent in the year 2014 on direct diabetes management in 2014, and complication costs were projected to be 25.7 billion from 2015 to 2025) (Mohan et al., 2020). On the other hand, 36.9% of Saudi Adults are obese and glycemic control is suboptimal in the majority (Nauck et al., 2021). In addition, GLP-1 agonists are associated with major adverse cardiovascular events reduction, therefore, GLP-1 agonists are considered in Saudi Arabia, liraglutide was approved in the year 2014, and semaglutide followed in the year 2020 to take the above-mentioned benefits. However, cost and access to treatments are major barriers to GLP-1 agonists use (Stegbauer et al., 2020; Alkhatib et al., 2022). In the present study, only 44.5% of patients who were prescribed GLP-1 agonists used them continuously, while 55.5% interrupted their treatment because of accessibility and cost. A plausible explanation could be that GLP-1 agonists are freely available for the treatment of patients with diabetes and obesity and not obesity without diabetes. Alkhatib et al. (Spinelli et al., 2025) found that semaglutide was the most cost-effective diabetes/obesity treatment, because of that the majority of our sample was on it followed by tirzepatide, while only a minority were on liraglutide injection. The uptake of GLP-1 agonists is relatively low (21.8%) compared to the United States of America in the off-label prescription of GLP-1 agonists ranging from 31.2% to 51.6% (Holland and Tiggemann, 2016). GLP-1 agonists in Saudi Arabia are higher compared to regional countries (12.3% in Jordan) (Börchers and Skibicka, 2025). The relatively low prescription rate raised awareness about the importance of health education in treating obesity as a chronic disease and the benefit of prescribing drugs with cardiac and renal protection for weight management.

We found an association between GLP-1 agonists, females, BMI, diet, and exercise; our findings were similar to Börchers and Skibicka (Pazzagli and Trolle, 2025) who observed the growing popularity in females with obesity. The associations between GLP-1 agonists with obesity, diet, and exercise were obvious because people with obesity are aware of the role of diet and exercise in weight management. The current findings of the association of GLP-1 use with women is similar to Pazzagli et al. (64) who found higher prescription of GLP-1 agonist in women compared to men.

## Study limitations

The study was limited by the reliance on a self-administered questionnaire and the study was conducted at a single city, because of that generalization cannot be ensured to the whole Kingdom of Saudi Arabia. In addition, selection bias from mall-based recruitment and recall bias from self-reported data significantly limited this study. Furthermore, we could not control for socioeconomic status in comparing GLP-1 agonists users and non-users, which may affect the validity of conclusions about smoking and antidepressant use.

## Conclusion

In the present study, GLP-1 agonists uptake was low despite the high rate of obesity. Importantly, the patients used GLP-1 agonists for weight reduction and not for comorbidities. In addition, more than half interrupted their intake due to cost/unavailability. Semaglutide was the most commonly prescribed medication followed by tirzepatide. Smoking was high, and antidepressant use was low compared to national data. No significant statistical difference between patients on/not on GLP-1 agonists regarding smoking, antidepressant use, and age, however, GLP-1 agonists use was associated with gender, BMI, diet, and exercise. Increasing the awareness of the general population that obesity is a chronic disease that needs long term treatment, the importance of healthy diet and exercise to maintain weight loss and reserve lean body mass and muscle mass, and adherence to GLP-1 agonists are highly recommended. Further, larger randomized control trials are needed to assess the different doses duration, and long-term effects of the recently introduced long-acting GLP-1 agonist and twincretins on smoking cessation and depression.

## Data Availability

The original contributions presented in the study are included in the article/supplementary material, further inquiries can be directed to the corresponding author.
